# The Incidence of Acute Peritonitis Secondary to Different Sites of Viscus Perforation

**DOI:** 10.7759/cureus.50479

**Published:** 2023-12-13

**Authors:** Hassan M Al Bisher, Hassan A Alsaleem, Arwa Althumairi, Ali H Almadan, Hussain Alaseel, Hassan S Alqattan, Mohammed K Alramadhan, Mahdi S Alabdullah, Faris M Al Matar, Sajjad M Almumen

**Affiliations:** 1 General Surgery, King Fahad University Hospital, Al Khobar, SAU; 2 Epidemiology and Public Health, Health Information Management and Technology (HIMT), Imam Abdulrahman Bin Faisal University, Dammam, SAU; 3 General Surgery, Imam Abdulrahman Bin Faisal University, Alqatif, SAU; 4 General Surgery, Imam Abdulrahman Bin Faisal University, Dammam, SAU

**Keywords:** large intestine, small intestine, comorbidities, viscus organ, peritonitis, perforation

## Abstract

Objective

The incidence of peritonitis secondary to viscus perforation will be examined to determine the most common sites of perforation and associated comorbidities.

Methods

This is a retrospective observational study based on data collected from the King Fahad Hospital of the University (KFUH). This research targeted patients who had viscus organ perforation and the relation of peritonitis secondary to it. The sample was taken from patients under the care of the General Surgery Department from the first of Feb 2016 to the 12th of Sep 2022. The final sample consisted of 450 patients. The method of diagnosis of peritonitis was mainly clinical, and the surgical approach was either through an exploratory laparotomy or a diagnostic laparoscopy. Incidental findings of viscus organ perforation were noted in addition to certain postoperative complications (e.g., adhesions) and hospital stay.

Results

Analysis of the results showed a significant relation (p<0.001) between viscus organ perforation and peritonitis. The most common comorbidities associated with secondary peritonitis were hypertension (12, 24.5%), diabetes mellitus (10, 20.4%), any abdominal mass (3, 6.1%), and inflammatory bowel disease (1, 2%). However, a chi-square analysis has shown no significant association between peritonitis and the targeted associated comorbidities.

Conclusion

Perforation of the small intestine carries the biggest association with peritonitis incidence, in addition to comorbidities such as hypertension and diabetes mellitus. Further study to establish the value of these factors might contribute to decreasing the morbidity and mortality of secondary peritonitis.

## Introduction

While medicine is a constantly developing field of science, there are always some diseases and conditions that affect it in terms of controlling morbidity and mortality. Peritonitis is one such disease. This affliction describes microbial contamination of the fluid present in the peritoneal sac, causing infection and inflammation. It can be classified into primary, secondary, and tertiary based on the source and nature of the microbial contamination. Primary peritonitis is an infection, often monomicrobial, of the peritoneal fluid without visceral perforation. Secondary peritonitis arises after the loss of integrity of a hollow viscus and is the most common form of peritonitis encountered, especially in developing countries such as India [1,2]. Tertiary peritonitis develops following treatment of secondary peritonitis either due to failure of the host inflammatory response or due to superinfection [3]. This microbial contamination of the peritoneal sac can develop into a sequence of infection, sepsis, multi-organ failure, and even death if not treated appropriately [4]. Even with proper treatment, the morbidity and mortality rates are still significant [5]. Hence, a more comprehensive understanding of this disease might aid in the production of a more favorable prognosis. This paper focuses on the most common sites of perforation that cause secondary peritonitis, and its most common associated comorbidities.

## Materials and methods

This is a retrospective observational study that was approved by the Institutional Review Board in King Fahad University Hospital (KFUH), Khobar, Saudi Arabia (IRB-UGS-2022-01-442). The data were collected through the KFUH system (QuadraMed; QuadraMed Corporation, Reston, Virginia) after acquiring authorization from the chairman of the General Surgery Department at the hospital. 

This research targeted patients who had hollow organ perforation and the relation of peritonitis secondary to the perforation. The sample was taken for patients under the care of the General Surgery Department in KFUH from the period between the first of Feb 2016 and the 12th of Sep 2022. A total of 500 patients who underwent either laparotomy or laparoscopic procedures were initially targeted, and 50 of these patients were excluded, leaving a total of 450 patients as the final sample. The exclusion criteria neglected patients under the age of one year.

The study consisted of various events that can result in perforation such as peptic ulcer, trauma, cholecystitis, diverticulosis, appendicitis, inflammatory bowel disease, malignancy, etc. Data collected included demographic data, incidence of peritonitis, sites of perforation, hospital stay time, and having one or more of the listed comorbidities (diabetes mellitus, hypertension, inflammatory bowel disease, or abdominal mass).

The diagnosis of secondary peritonitis was clinical, in addition to radiological and laboratory findings that supported the diagnosis of peritonitis or perforation. The surgical approach for these patients was either exploratory laparotomy or diagnostic laparoscopy. Incidental findings of perforation in organs such as large bowel, small bowel, stomach, appendix, gallbladder, etc. were identified during the procedures. Postoperative complications, including bleeding, wound infection, perforation of adjacent organs, adhesion, and obstruction, were noted. Other surgical complications were not considered, such as nerve damage or organ failure. Additionally, postoperative hospital stay was compared between patients.

The goal of this research is to figure out the most common sites of perforation causing peritonitis. In addition, different variables or diseases that can predispose to peritonitis or perforation were considered, and these included diabetes mellitus, hypertension inflammatory bowel disease, and the presence of an abdominal mass.

All analysis was performed using Statistical Product and Service Solutions (SPSS) (IBM SPSS Statistics for Windows, Armonk, NY). Categorical variables were reported as frequencies and percentages. Chi-square was used to assess the association between peritonitis and perforation. The significance value set at p>0.05 was significant.

## Results

In this study, 450 patients are targeted. Most cases were male 279 (62%), while female cases were 171 (38%). The peak incidence of cases was observed in patients in the fourth decade. The average length of stay in the hospital was 25 days. There was no significant difference in sex and length of stay between patients with peritonitis and those without peritonitis. In Table 1, it was found through a chi-square test that the only patient factor that determines the presence of peritonitis is the presence of perforation, while the other patient factors do not influence peritonitis incidence.

**Table 1:** Relation between patient characteristics and peritonitis. SD: standard deviation; N: number of patients

Patient characteristics		Peritonitis		All patient	Chi-square test	P- value
		Yes N=49 (10.8%)	No N=401 (89.1%)			
Sex	Male	29 (10.4%)	250 (89.6)	279 (62)	185	0.667
	Female	20 (11.7%)	151 (88.3%)	171 (38%)		
Age (Mean (SD))		44 (21%)	40 (22%)	40 (22%)	-1	0.238
Length of Stay (Mean (SD)		27 (39%)	24 (42%)	25 (41%)	0	0.701
Perforation	No	16 (5.1%)	296 (94.9%)	312 (69.3%)	34.795	<0.001
	Yes	33 (23.9%)	105 (76.1%)	138(30.7%)		

The most common sites of perforation encountered in patients are the small intestine (75, 56.3%), followed by the colon (34, 25%), stomach (15, 11.2%), appendix (six, 4.5%), gallbladder (one, 0.75%), urinary bladder (one, 0.75%), and uterus (one, 0.75%). However, the most common site of perforation associated with peritonitis is the small intestine (15, 45%) (Table 2). There was no significant association between the number of patients with peritonitis and the type of comorbidities encountered.

**Table 2:** Common site of perforation. N: Number of patients

Perforation/peritonitis	Site of perforation							Chi-square test	P-value
Perforation	Appendix	Colon	Gall Bladder	Intestine	Stomach	Bladder	Uterus		
N (%) with perforation	6 (4.5%)	34 (25%)	1 (0.75%)	75 (56.3%)	15 (11.2%)	1 (0.75%)	1 (0.75%)	426.855	<0.001
Peritonitis & perforation, N (%)	2 (6.1%)	14 (42.4%)	0 (0%)	15 (45.5%)	2 (6.1%)	0 (0%)	0 (0%)	53.282	<0.001

There was no significant association between the number of patients with peritonitis and the type of comorbidities encountered. Table 3 shows that the most common diseases associated with peritonitis patients are hypertension (12, 24.5%), followed by diabetes mellitus (10, 20.4%), any abdominal mass (three, 6.1%), and inflammatory bowel disease (one, 2%).

**Table 3:** Relation between the types of comorbidities and peritonitis. N: Number of patients; DM: Diabetes mellitus; HTN: Hypertension; IBD: Inflammatory bowel disease. Have comorbidity: a patient who has one or more diseases of diabetes mellitus, hypertension, inflammatory bowel disease, or any mass in the abdomen.

Type of comorbidity		Peritonitis				Chi-square test	P-value
		No		Yes			
		N	%	N	%		
DM	No	335	83.5%	39	79.6%	0.485	0.486
	Yes	66	16.5%	10	20.4%		
HTN	No	318	79.3%	37	75.5%	0.377	0.539
	Yes	83	20.7%	12	24.5%		
IBD	No	377	88.7%	48	98%	1.295	0.255
	Yes	24	6%	1	2%		
Any mass in the abdomen	No	370	88.9%	46	93.9%	0.162	0.688
	Yes	31	7.7%	3	6.1%		
Have comorbidity	No	254	63.3%	33	67.3%	0.303	0.582
	Yes	147	36.7%	16	32.7%		

There was no significant association between the number of patients with perforation and the type of comorbidities encountered. Table 4 shows that the most common diseases associated with perforated patients are hypertension (34, 24.6%), followed by diabetes mellitus (25, 18.1%), inflammatory bowel disease (five, 3.6%), abdominal mass that includes the cecum, rectum, and small intestine (four, 2.9%).

**Table 4:** Relation between the type of comorbidities and perforation. N: Number of patients; DM: Diabetes mellitus; HTN: Hypertension; IBD: Inflammatory bowel disease. Have comorbidity: a patient who has one or more diseases of diabetes mellitus, hypertension, inflammatory bowel disease, or any mass in the abdomen.

Type of comorbidity		Perforation				Chi-square test	P-value
		No		Yes			
		N	%	N	%		
DM	No	261	83.7%	113	81.9%	0.214	0.644
	Yes	51	16.3%	25	18.1%		
HTN	No	251	80.4%	104	75.4%	1.486	0.223
	Yes	61	19.6%	34	24.6%		
IBD	No	292	93.6%	133	96.4%	1.416	0.234
	Yes	20	6.4%	5	3.6%		
Any mass in the abdomen	No	282	90.4%	134	97.1%	6.180	0.13
	Yes	30	9.6%	4	2.9%		
Have comorbidity	No	195	62.5%	92	66.7	0.719	0.396
	Yes	117	37%	46	33.3%		

## Discussion

Perforation could occur in different sites inside the body involving a variety of hollow viscus structures. It can be a result of different factors, causing several noticed outcomes, including peritonitis. Perforating peritonitis is mostly the result of perforation of a diseased viscus [6]. It is the most common surgical emergency in general surgery, which is perforation peritonitis [1,7-9]. The mortality rate can be influenced by the anatomical site of perforation as it can also influence the source of normal flora, and the highest rates in several studies tend to be large bowel sources [10,11].

In this study, 62% of the patients involved were males, and 38% were females in comparison to that of Fallani et al.'s study, wherein participants' ratio was 53% males and 47% females. Moreover, 10.4% of all males and 11.7% of all females in the current study had peritonitis [12]. Furthermore, the mean age of patients with peritonitis was 44 years old in both studies [12]. In Eastern studies, the male-to-female ratio in perforation peritonitis tends to be higher in males, which is thought to be more due to alcoholism and smoking [1,8-10].

In this research, patients who had perforation were 138 out of the 450 patients who were selected. The most common sites of perforation were the small intestine (56.3%), followed by the colon (25%), stomach (11.2%), appendix (4.5%), urinary bladder (0.75%), gallbladder (0.75%), and lastly the uterus (0.75%). These results were compared to the study done by Poudel et al., which demonstrated that the most common sites were prepyloric perforations, followed by duodenal perforations [13]. According to Afridi et al., the first place was the duodenum (43.6%), ileum (37.6%), and lastly the colon (8%) [14]. When it comes to the cause, it can differ for the site of perforation; for example, in typhoid fever and tuberculosis, the site of perforation tends to be distal ileal perforation [1,10]. In trauma perforation, the jejunum is usually the most common site [1,11]. In peptic ulcers, the most common site is the first part of the duodenum [1,7,9,11]. The site of perforation in Eastern countries such as India and Pakistan tends to be proximal gastrointestinal origin, whereas in Western countries, it tends to be distal gastrointestinal [1,7].

Comorbidities in this research were taken into consideration for all assessed patients. The most common diseases associated with secondary peritonitis are hypertension (24.5%), diabetes mellitus (20.4%), abdominal masses (6.1%), and inflammatory bowel diseases (2%). In comparison to relevant research, it was found that hypertension was the most prevalent associated morbidity, reaching 58.8% of secondary peritonitis patients, followed by diabetes mellitus (29.4%) and malignancy (14.7%) [15]. Various factors are associated with morbidity and mortality from delay of intervention, postoperative complications, and existing comorbidities [1,7]. Colorectal perforation tends to carry the highest complication rates, which is unsurprising as the bacterial content can arouse bacterial peritonitis [10,11].

The average length of hospital stay for participants who had peritonitis was 27 days, while the average for participants who did not was 24 days. This can be attributed to multiple factors as comorbidities, complications, and peritonitis. The main source of treatment is adequate resuscitation, antibiotics, surgical interventions to ensure pathological progress, and eliminating the source of contamination [7,10]. Perforation, if left untreated, can evolve into sepsis and peritonitis [11]. Septicemia is usually the main cause of mortality in most cases [7-9], and wound infection is the most common postoperative complication [7,9].

Application

Peritonitis secondary to viscus perforation is a commonly encountered emergency in the operation room. A further understanding of this problem would aid greatly in its treatment and prognosis.

Limitations and recommendations

There were a couple of limitations encountered during the study. A notable one would be that the data were taken from a single center. Another one worth mentioning is that there was an inability to accurately pinpoint the site of perforation when the defect occurred in the small intestine. Moreover, the etiology of perforation and indication of surgeries were not studied thoroughly. In addition, the significance of the surgical approach was not noted. If the study were to be expanded, it would benefit greatly from conducting it in a multi-center experience or even nationwide. Furthermore, accuracy in determining the site of perforation in the small intestine would aid in the prediction of the defect site in a clinical setting.

## Conclusions

Peritonitis and dealing with its complications, especially in patients with multiple comorbidities, require an approach that considers time-sensitive management. Any progress that assists in the early initiation of treatment would be of huge utility in achieving a favorable prognosis. If the study is to be expanded, it would benefit greatly from conducting it in a multi-center experience or even nationwide. In addition, accuracy in determining the site of perforation in the small intestine would aid in the prediction of the defect site in a clinical setting.

## References

[1] Hameed T, Kumar A, Sahni S, Bhatia R, Vidhyarthy AK (2020). Emerging spectrum of perforation peritonitis in developing world. Front Surg.

[2] Sharma Sharma, Kaneria Kaneria, Sharma Sharma (2019). Perforation peritonitis: a clinical study regarding etiology, clinical presentation and management strategies. Int Surg J.

[3] Simmen HP, Heinzelmann M, Largiadèr F (1996). Peritonitis: classification and causes. Dig Surg.

[4] Gupta S, Kaushik R, Kaur A, Attri AK (2006). Tubercular appendicitis - a case report. World J Emerg Surg.

[5] Tochie JN, Agbor NV, Frank Leonel TT, Mbonda A, Aji Abang D, Danwang C (2020). Global epidemiology of acute generalised peritonitis: a protocol for a systematic review and meta-analysis. BMJ Open.

[6] Chakma SM, Singh RL, Parmekar MV (2013). Spectrum of perforation peritonitis. J Clin Diagn Res.

[7] Neupane S, Koirala DP, Kharel S, Silwal S, Yadav KK (2022). Clinical profile and management of perforation peritonitis in Bharatpur hospital, Nepal: a prospective study. Ann Med Surg (Lond).

[8] Jhobta RS, Attri AK, Kaushik R, Sharma R, Jhobta A (2006). Spectrum of perforation peritonitis in India - review of 504 consecutive cases. World J Emerg Surg.

[9] Kallely MF, Panchabhai SV, Nichkaode PB, Rayani HP, Ravi Teja JC, Patil DA (2020). Perforation peritonitis: a clinical profile and management. SLJS.

[10] Lohith P, Jindal RK, Ghuliani D, Rajshekar P (2020). The anatomical site of perforation peritonitis and their microbiological profile: a cross-sectional study. Int Surg J.

[11] Pouli S, Kozana A, Papakitsou I, Daskalogiannaki M, Raissaki M (2020). Gastrointestinal perforation: clinical and MDCT clues for identification of aetiology. Insights into Imaging.

[12] Fallani G, Lombardi R, Masetti M (2021). Urgent and emergency surgery for secondary peritonitis during the COVID-19 outbreak: an unseen burden of a healthcare crisis. Updates Surg.

[13] Poudel R, Shah S, Chandra K, Pradhan S, Joshi P (2018). Spectrum of perforation peritonitis in western Nepal. J Univers Coll Med Sci.

[14] Afridi SP, Malik F, Ur-Rahman S, Shamim S, Samo KA (2008). Spectrum of perforation peritonitis in Pakistan: 300 cases Eastern experience. World J Emerg Surg.

[15] Doklestić S, Bajec D, Djukić R (2014). Secondary peritonitis - evaluation of 204 cases and literature review. J Med Life.

